# Photothermal therapy improves the efficacy of a MEK inhibitor in neurofibromatosis type 1-associated malignant peripheral nerve sheath tumors

**DOI:** 10.1038/srep37035

**Published:** 2016-11-11

**Authors:** Elizabeth E. Sweeney, Rachel A. Burga, Chaoyang Li, Yuan Zhu, Rohan Fernandes

**Affiliations:** 1 The Sheikh Zayed Institute for Pediatric Surgical Innovation, Children’s National Health System, 111 Michigan Ave NW, Washington, DC 20010, USA; 2 Institute for Biomedical Sciences, The George Washington University, 2330 Eye St NW, Washington, DC 20037, USA; 3 Center for Cancer and Immunology Research, Children’s National Health System, 111 Michigan Ave NW, Washington, DC 20010, USA; 4 Department of Radiology, The George Washington University, 2330 Eye St NW, Washington, DC 20037, USA; 5 Department of Pediatrics, The George Washington University, 2330 Eye St NW, Washington, DC 20037, USA

## Abstract

Malignant peripheral nerve sheath tumors (MPNSTs) are aggressive tumors with low survival rates and the leading cause of death in neurofibromatosis type 1 (NF1) patients under 40 years old. Surgical resection is the standard of care for MPNSTs, but is often incomplete and can generate loss of function, necessitating the development of novel treatment methods for this patient population. Here, we describe a novel combination therapy comprising MEK inhibition and nanoparticle-based photothermal therapy (PTT) for MPNSTs. MEK inhibitors block activity driven by Ras, an oncogene constitutively activated in NF1-associated MPNSTs, while PTT serves as a minimally invasive method to ablate cancer cells. Our rationale for combining these seemingly disparate techniques for MPNSTs is based on several reports demonstrating the efficacy of *systemic* chemotherapy with *local* PTT. We combine the MEK inhibitor, PD-0325901 (PD901), with Prussian blue nanoparticles (PBNPs) as PTT agents, to block MEK activity and simultaneously ablate MPNSTs. Our data demonstrate the synergistic effect of combining PD901 with PBNP-based PTT, which converge through the Ras pathway to generate apoptosis, necrosis, and decreased proliferation, thereby mitigating tumor growth and increasing survival of MPNST-bearing animals. Our results suggest the potential of this novel local-systemic combination “nanochemotherapy” for treating patients with MPNSTs.

Neurofibromatosis type 1 (NF1) is a disorder of the nervous system affecting 1 in ~3500 individuals worldwide^1^,^2^. This disorder is characterized by the development of benign neurofibromas, a significant portion of which progresses to malignant peripheral nerve sheath tumors (MPNSTs), aggressive tumors with low 5-year survival rates (<50%) and the leading cause of death in NF1 patients under 40 years old^2^,^3^. Surgical resection is the standard of care for MPNSTs^4^. However, surgery can be invasive, debilitating, incomplete, and result in loss of function^5^. This necessitates the development of novel methods for the management of MPNSTs. In response to this need, we describe a novel combination therapy of systemically (orally) administered MEK inhibitors with locally (intratumorally) administered nanoparticle-based photothermal therapy (PTT) for treating MPNSTs.

Our rationale for combining MEK inhibition with PTT is premised on precedent in the literature that has demonstrated the improved efficacy of combining chemotherapy with PTT for treating diverse cancers^6^,^7^,^8^,^9^,^10^,^11^,^12^,^13^,^14^. Studies have successfully used graphene oxide^8^, gold nanorods^10^, and nanoshells^13^ as agents for PTT to improve the efficacy of chemotherapy in cancers such as inflammatory breast cancer^13^ and hepatocellular carcinoma^12^. One mechanism by which PTT improves the efficacy of chemotherapy is by increasing the membrane permeability of targeted tumor cells causing increased uptake of the chemotherapeutic agent^13^. Conversely, PTT also benefits from chemotherapy, which elicits systemic effects to complement its inherently local effects. Motivated by these earlier findings, we seek to exploit these complementary effects in the context of NF1-associated MPNSTs. Specifically, we combine the MEK inhibitor, PD-0325901 (PD901), with Prussian blue nanoparticles (PBNPs) as PTT agents, to block MEK activity and simultaneously ablate MPNSTs when irradiated with a near infrared (NIR) laser. To our knowledge, our study represents the first attempt at exploiting the synergy between PTT and chemotherapy for the treatment of NF1-associated MPNSTs.

MEK inhibitors are small molecule inhibitors that target the Ras signaling pathway. NF1 and NF1-associated MPNST patients pathognomonically lack neurofibromin, a negative regulator of oncogenic Ras signaling. Without neurofibromin protein function, Ras is allowed constitutive activation^15^,^16^. The Ras signal transduction pathway generates a phosphorylation cascade through RAF, MEK, and ERK, which in its phosphorylated form (p-ERK) affects the transcription of genes associated with uncontrolled cell proliferation and increased cancer progression^17^,^18^. Research suggests the potential of using MEK inhibitors to block Ras activity in MPNSTs^19^,^20^,^21^,^22^, but these studies were conducted in either cell lines^19^,^21^,^22^ or in animal models that yielded marginal results in treating MPNSTs^20^,^23^. Based on the improved efficacy of combining chemotherapy with PTT, we expect that the effects of the MEK inhibitor PD901 will be made more potent when combined with PBNP-based PTT for treating MPNSTs. PTT is a minimally invasive method for destroying tumors using light-activated nanoparticles and a low power NIR laser^24^,^25^. In this study, we use PBNPs^26^,^27^,^28^,^29^ for PTT of MPNSTs, which we have previously used for ablation of subcutaneous neuroblastoma^28^. Compared to alternative nanoparticles used for PTT, PBNPs offer several advantages: they can easily be synthesized in a single, scalable step at low costs, and are already FDA-approved for human oral consumption (to treat radioactive poisoning)^30^,^31^ suggesting their potential safety for use as PTT agents.

To determine whether PD901 combined with PBNP-based PTT results in improved treatment outcomes for MPNSTs, we use the mouse M2 MPNST cells *in vitro*, and a syngeneic, subcutaneous mouse model of MPNST *in vivo*^32^. First, we assess the efficacy of PD901 and PBNP-based PTT individually in treating MPNST cells *in vitro*. Specifically, we assess the effects of the individual therapies on MPNST cell viability and the mechanisms of cell death induced by the therapies. Next, we assess the efficacy of the PD901/PTT combination in treating MPNST cells *in vitro* and whether this combination of PD901 and PTT is synergistic (using dose reponse and drug interaction calculations). Finally, we determine the effects of the PD901/PTT combination on tumor progression and animal survival *in vivo*. The findings of these studies will demonstrate the feasibility of using our novel nanochemotherapy for preclinically treating MPNSTs, an important prelude to eventual clinical translation.

## Results and Discussion

### PD901 effectively treats MPNSTs *in vitro* by blocking ERK activation

**Figure 1:**
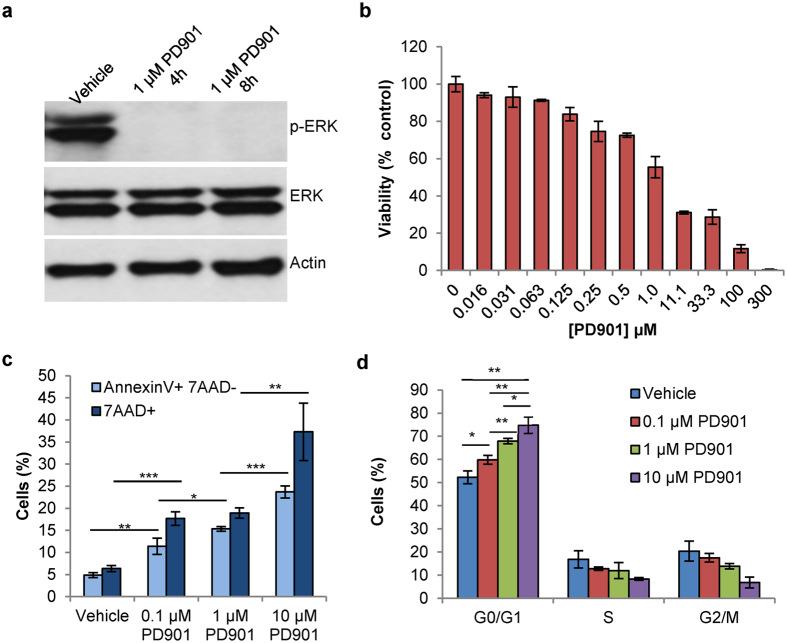
PD901 effectively treats MPNSTs *in vitro* by blocking ERK activation. (**a**) Mouse MPNST (M2) cells exhibit markedly decreased p-ERK protein expression when treated with 1 μM PD901 for 4 h or 8 h compared to vehicle (DMSO)-treated M2 cells when visualized by a Western blot. (**b**) M2 cells exhibit decreased viability when treated for 48 h with increasing concentrations of PD901 (IC50 = 1 μM). (**c**) M2 cells treated with 0.1, 1, and 10 μM PD901 for 24 h undergo cell death via both apoptosis (light blue, Annexin V + 7AAD- population) and late apoptosis/necrosis (dark blue, 7AAD + population), measured by flow cytometry. (**d**) M2 cells treated with 0.1, 1, and 10 μM PD901 for 24 h undergo cell cycle arrest in the G0/G1 phase in a concentration-dependent manner, measured by flow cytometry. Data in all plots expressed as mean ± standard deviation (n ≥ 3/group); *p < 0.05; **p < 0.01; ***p < 0.001.

In order to validate the presumed anti-MEK mechanism of action of PD901 in M2 cells, we measured its effects on the activation of ERK, which is located downstream of MEK in the Ras signaling pathway. M2 cells were treated with vehicle (DMSO) or 1 μM PD901 for four or eight hours, and then harvested, lysed, and probed for phosphorylated ERK (p-ERK) and total ERK, using actin as a loading control. 1 μM PD901 effectively blocked the activation of ERK (p-ERK) at both four and eight hours, demonstrated by no visible p-ERK bands on the Western blot in the PD901-treated lanes, compared with distinct p-ERK bands observed in the vehicle-treated lane (Fig. 1a). Our finding of decreased p-ERK expression in M2 cells is consistent with those in the published literature, which confirm decreased ERK activation after a few hours of PD901 treatment^33^. To determine the effect of MEK inhibition on MPNST viability, we treated M2 cells with varying doses of PD901 and measured cell viability. PD901 decreased M2 cell viability in a concentration-dependent manner, with an IC50 of approximately 1 μM (Fig. 1b). These results are consistent with earlier studies using MEK inhibitors in other cell lines^23^,^34^. To determine the mechanisms causing decreased M2 viability, we analyzed the mechanisms of cell death (apoptosis versus late apoptosis/necrosis), and the effects of PD901 treatment on the M2 cell cycle. PD901 was found to decrease M2 viability by both apoptosis (Annexin V + 7AAD-) and late apoptosis/necrosis (7AAD+) at all concentrations tested (Fig. 1c). Further, PD901 caused a significant arrest of M2 cells in the G0/G1 phase in a concentration-dependent manner (Fig. 1d), indicating decreased proliferation after treatment. Our results with M2 cells suggest that MEK inhibition using PD901 is a feasible modality for treating MPNST *in vitro*, by the blockade of ERK activation, which would otherwise increase cell proliferation and division^2^.

### PBNPs function as effective agents for PTT of MPNSTs *in vitro*

**Figure 2:**
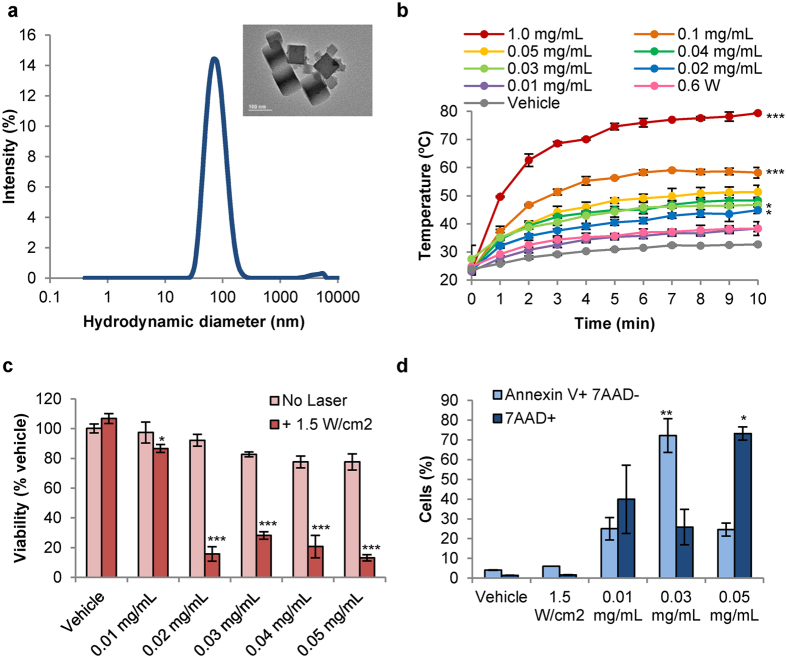
PBNPs function as effective agents for PTT of MPNSTs *in vitro*. (**a**) PBNPs exhibit monodisperse size distributions as measured by dynamic light scattering (mean hydrodynamic diameter = 68.1 nm). Inset: PBNPs exhibit their characteristic cubic morphology when visualized using transmission electron microscopy (scale bar = 100 nm). (**b**) PBNPs heat to higher temperatures with increasing concentrations of PBNPs when irradiated with an 808 nm NIR laser at 1.5 W/cm^2^ for ten minutes; measured at one-minute intervals using a thermocouple. *p < 0.05; ***p < 0.001; compared to adjacent lower concentration’s temperature at 10 minutes. (**c**) The viability of M2 cells decrease when subject to varying doses of PTT (PBNP concentrations ranging from 0.01 mg/mL to 0.05 mg/mL, with or without 808 nm laser irradiation at 1.5 W/cm^2^ for ten minutes), measured after 24 h. *p < 0.05; ***p < 0.001; compared to matched controls. (**d**) M2 cells treated with various doses of PTT can trigger cell death via apoptosis (light blue, Annexin V + 7AAD- population) and/or late apoptosis/necrosis (dark blue, 7AAD + population) depending on the resulting temperature range to which they are heated; quantified by flow cytometry. Data in all plots expressed as mean ± standard deviation (n ≥ 3/group). *p < 0.05; **p < 0.01; compared to all other samples in group.

To determine whether PBNPs function as effective agents for PTT of MPNSTs, we conducted studies *in vitro* using M2 cells. First, PBNPs were synthesized with monodisperse size distributions as measured by dynamic light scattering (mean hydrodynamic diameter of 68.1 nm), and with their characteristic cubic structures as observed by transmission electron microscopy (Fig. 2a). The synthesized PBNPs heated in a concentration-dependent manner when irradiated with an 808 nm NIR laser at 1.5 W/cm^2^ laser power density for 10 minutes (Fig. 2b). Importantly, this incremental heating consistently resulted in decreased viability of M2 cells when the PBNPs were similarly irradiated with the NIR laser (Fig. 2c). Temperatures around 45 °C and greater appeared to decrease viability of M2 cells to a similar extent (approximately 15–25% viable). Interestingly, PBNP-based PTT triggered differing levels of apoptosis and/or late apoptosis/necrosis in M2 cells depending on the temperature ranges to which they were heated. This temperature range was, in turn, dependent on the concentration of PBNPs used for PTT since both laser power and duration of laser irradiation were kept constant in these studies. M2 cells that were heated to the 45–50 °C temperature range triggered cell death through primarily apoptosis; M2 cells treated with 0.03 mg/mL PBNPs, which attained an average temperature of 46.8 °C after ten minutes irradiation at 1.5 W/cm^2^, resulted in apoptosis in 72.2% of cells. In contrast, M2 cells that were heated to >50 °C triggered cell death through primarily late apoptosis/necrosis; M2 cells treated with 0.05 mg/mL PBNPs, which attained an average temperature of 51.3 °C after ten minutes irradiation at 1.5 W/cm^2^, resulted in late apoptosis/necrosis in 73.2% of cells (Fig. 2d). Our finding of a temperature range preferentially triggering cell death by either apoptosis or necrosis are corroborated by an earlier study using gold nanorods to ablate tumor cells *in vitro*^35^. Thus, PBNPs function as effective agents for PTT of MPNSTs resulting in decreased viability of these cells *in vitro*. More importantly, our data suggests a potentially tunable mechanism of inducing cell death using PBNP-based PTT (apoptosis versus necrosis), which can be leveraged in subsequent studies to improve treatment outcomes for MPNSTs *in vivo*.

### PD901 and PTT synergistically combine to yield improved treatment outcomes for MPNSTs *in vitro*

**Figure 3:**
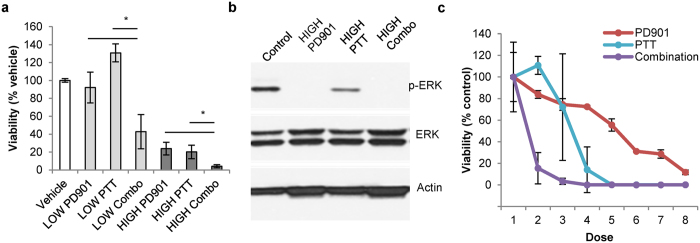
PD901 and PTT synergistically combine to yield improved treatment outcomes for MPNSTs *in vitro*. (**a**) M2 cells treated with low and high doses of PD901 and PTT show significantly decreased cell viability in combination therapy (PD901 plus PTT)-treated groups relative to either therapy administered individually under identical conditions after 24 h. LOW PD901: 0.125 μM; LOW PTT 0.005 mg/mL + 1.5 W/cm^2^ 808 nm laser for 10 minutes; LOW Combo: both LOW PD901 + LOW PTT treatments; HIGH PD901: 1.0 μM; HIGH PTT 0.05 mg/mL + 1.5 W/cm^2^ 808 nm laser for 10 minutes; HIGH Combo: both HIGH PD901 + HIGH PTT treatments. Data expressed as mean ± standard deviation (n ≥ 3/group). *p < 0.05. (**b**) M2 cells treated with vehicle, HIGH PD901, HIGH PTT, HIGH Combo for 4 h, and subsequently harvested, lysed, and probed for p-ERK, exhibit complete eradication of p-ERK expression in both the PD901 and HIGH Combo groups, and a clear decrease in the HIGH PTT-treated group. Actin was used as a loading control. (**c**) PD901 synergistically combines with PTT to decrease M2 cell viability *in vitro* over either treatment alone at equivalent doses. 50,000 M2 cells were treated with increasing doses of PD901, PTT, or combination, and viability was measured after 24 h by CellTiter-Glo viability assay (Promega). Data points represent means ± standard deviation; n = 3 per group.

After determining the individual efficacies of both PD901 and PBNP-based PTT in treating M2 cells *in vitro,* we next sought to examine the potential benefit of combining the treatments. Cell viability studies demonstrated that combining the two treatments *in vitro* was able to significantly decrease the viability of M2 cells more than either treatment administered alone (Fig. 3a). At both low doses (0.125 μM PD901, “LOW PD901;” 0.005 mg/mL PBNP-based PTT, “LOW PTT”) and high doses (1 μM PD901, “HIGH PD901;” 0.05 mg/mL PBNP-based PTT, “HIGH PTT”), the combination treatment resulted in significantly decreased cell viability over the individual treatments administered under identical conditions. Additionally, PD901 + PTT (“HIGH Combo”) functioned to effectively block p-ERK expression in M2 cells, mirroring the effects of PD901 treatment alone (Fig. 3b). It is important to note that while both PD901 alone and the HIGH Combo treatment resulted in similar Western blot readouts, further studies are needed to elucidate whether these effects are sustained in the two treatment groups over time. Earlier studies have demonstrated a rescue of p-ERK expression over time after MEK inhibition^23^. Therefore, therapies that yield sustained decrease of p-ERK expression may be important for improved treatment outcomes in MPNSTs. Interestingly, PTT alone was able to decrease p-ERK expression (Fig. 3b), signifying its potential impact on Ras signaling at a point along its signal transduction pathway. This finding suggests that although PD901 and PTT function differently to decrease M2 cell viability (cell cycle arrest vs. ablation, respectively), these effects converge along the Ras signaling pathway. To our knowledge, using PTT to alter a prominent signal transduction pathway in cancer has not been previously explored. Deeper mechanistic studies to determine the effect of PTT on Ras signaling in the context of NF1-associated MPNST are ongoing. As a final component of our studies assessing the effects of the PD901/PTT combination *in vitro*, we sought to determine if the effects of PD901 and PTT on decreasing M2 cell viability are synergistic. Dose response curves measuring the viability of M2 cells *in vitro* in response to increasing concentrations of either individual treatment or the PD901/PTT combination demonstrated that PD901 and PTT synergistically combine to decrease M2 viability (Fig. 3c). The coefficients of drug interaction (CDI)^36^ for three of the doses tested (doses 2, 3, and 4) were calculated to be less than 0.7, indicating significant drug synergy between PD901 and PTT.

### PD901 and PTT combine to decrease MPNST progression and increase survival *in vivo*

**Figure 4:**
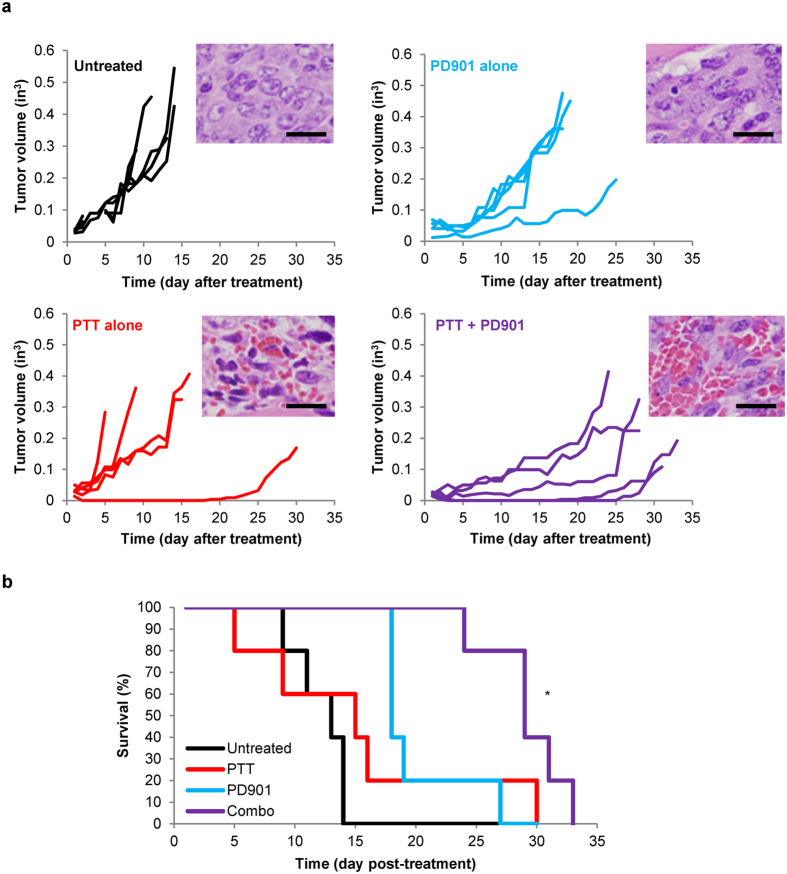
PD901 and PTT combine to decrease MPNST progression and increase survival *in vivo*. MPNST-bearing B6129SF1/J mice were treated once tumors reached 10 mm in diameter with: no treatment (n = 5, black), 5 mg/kg PD901 (n = 5, blue), PTT (n = 5, red; 1.0 mg/mL PBNPs with 1.5 W/cm^2^), or PD901 + PTT (n = 5, purple; both PD901 and PTT treatments). PD901 was administered by oral gavage daily. PBNPs were administered intratumorally and PTT was performed once for ten minutes. Animals were euthanized when tumors reached 20 mm in diameter or showed signs of distress. (**a**) Tumor progression was measured every day by calipers. Each line represents one mouse. Insets: Tumors were harvested 8 h post-treatment, processed for histology, stained with H&E, and visualized by microscopy; scale bar = 20 μm. (**b**) Survival is illustrated by a Kaplan-Meier curve. *p < 0.05 compared to all groups by log-rank test.

Motivated by our *in vitro* findings, we generated a syngeneic mouse model of MPNST using the M2 cell line in B6129SF1/J mice based on previous literature^32^ to test the *in vivo* efficacy of our novel combination therapy. Mice were subcutaneously injected with M2 cells. When the mice exhibited established tumors (~10 mm in diameter), the MPNST-bearing mice were divided into four groups and were: 1) untreated (n = 5), 2) treated with PD901 (n = 5), 3) treated with PTT (n = 5), or 4) treated with both PD901 plus PTT (n = 5). Tumor progression was measured daily and mouse survival was monitored post-treatment. Tumors in the untreated group progressed at the fastest rate (Fig. 4a, black lines), and mice in this group succumbed to high tumor burden after 14 days or less (median survival = 13 days; Fig. 4b). In contrast, mice treated with PD901 plus PTT exhibited slower tumor growth compared to all other groups, as demonstrated by the decreased slopes of their tumor progression curves (Fig. 4a, purple lines). This slower tumor growth translated to significantly increased survival in mice treated with both PD901 plus PTT (median survival = 29 days) compared to mice in all other treatment groups, where median survivals of 18 days and 15 days were observed in PD901-treated and PTT-treated mice, respectively (Fig. 4b; p < 0.05). Hematoxylin and eosin (H&E) stains of tumors extracted from MPNST-bearing mice 8 h after treatment confirmed these findings of increased tumor cell death post-combination treatment (Fig. 4a and Supplementary Figure S1). It should be noted that the improved treatment outcomes were observed using a single local administration of PBNP-based PTT and daily oral dosing of PD901. Further optimization of this combination therapy that leverages the convergent biological effects of these two therapies observed *in vitro* may result in further improvement in treatment outcomes for MPNSTs over those currently observed, in addition to conferring potential benefits such as less frequent doses of the MEK inhibitor. Taken together, our findings demonstrate the feasibility of using PD901 in combination with PBNP-based PTT for achieving improved treatment outcomes for MPNSTs *in vivo*.

In summary, we have presented a novel combination therapy comprising two distinct but synergistic treatments: MEK inhibition using the small molecule inhibitor PD901 and PBNP-based PTT. We demonstrated a convergence of the two treatment strategies on the Ras signal transduction pathway, which resulted in decreased ERK activation. These effects, when combined with the effects of photothermal ablation via PTT, resulted in decreased growth of MPNSTs both *in vitro* and *in vivo.* Ongoing studies in our group will build upon these data to further mechanistically describe the combined effect of PD901 and PTT so as to better exploit the synergy of this combination nanochemotherapy in clinically treating NF1-associated MPNSTs.

## Methods

### Chemicals and PBNP synthesis

PD0325901 (#PZ0162; PD901) was purchased from Sigma-Aldrich (St. Louis, MO). PBNPs were synthesized from their constituent salts purchased from Sigma-Aldrich as previously described^28^.

### MPNST cells

The M2 mouse MPNST cell line was a gift from Dr. Samuel A. Rabkin (Massachusetts General Hospital, Harvard Medical School; Boston, Massachusetts)^32^, originally isolated from spontaneously arising tumors in Nf1/Trp53 heterozygous mice obtained from the LF Parada laboratory. The M2 cells were cultured in DMEM with 10% FBS and 1% Penicillin/Streptavidin (Life Technologies, Carlsbad, CA).

### Western blots

Treated M2 cells were lysed in 1X RIPA buffer [50 mM Tris-HCL (pH 7.4), 150 mM NaCl, 1% Triton X-100, 1% sodium deoxycholate, 0.1% SDS, 1 mM EDTA, complete protease inhibitor (Roche, Basel, Switzerland) and phosphatase inhibitor (Roche, Basel, Switzerland)]. Samples were analyzed by SDS-PAGE and transferred onto PVDF membranes (Millipore, Billerica, MA). The blots were then blocked in 5% non-fat milk in TBST, followed by incubation of primary antibodies at 4 °C overnight. After washing, the blots were incubated in horseradish peroxidase (HRP)-conjugated secondary antibodies at room temperature for 1 hour. Signals were detected using ECL or ECL plus (GE healthcare, Little Chalfont, United Kingdom) followed by film development. The primary antibodies used are as follows: p-ERK (1:1,000, rabbit, Cell Signaling, Danvers, MA), ERK (1:2,000, rabbit, Cell Signaling, Danvers, MA), and β-actin (1:10,000, mouse, Sigma-Aldrich, St. Louis, MO).

### Cell viability assays

M2 cells were seeded at 50,000 cells per well in 96-well cell culture plates overnight. After treatment, the cells were analyzed for viability using the ATP-based CellTiter-Glo Luminescent Cell Viability Assay (Promega, Madison, WI). Luminescence was read on the EnSpire Multimode Plate Reader (PerkinElmer, Waltham, MA).

### Flow cytometry

After the relevant treatments, M2 cells were stained with PE-Annexin V (BD Pharmingen, Franklin Lakes, NJ) and 7AAD (BD Pharmingen, Franklin Lakes, NJ). For cell cycle analysis, M2 cells were stained with propidium iodide (ThermoFisher Scientific, Carlsbad, CA) after treatment. Flow cytometry was performed on a BD FACSCalibur and analyzed with the FlowJo 7.6 software.

### Dynamic light scattering

Hydrodynamic diameter of PBNPs (10 μg/mL) was measured by dynamic light scattering on a Zetasizer (Malvern Instruments Ltd., Malvern, United Kingdom) as per the manufacturer’s specifications.

### Transmission electron microscopy

PBNPs were dropped onto 100 mesh standard formvar grids (Electron Microscopy Sciences, Hatfield, PA) and visualized by transmission electron microscopy on a JEM-2100 FEG high-resolution transmission electron microscope at 200 kV.

### Photothermal therapy

An 808 nm NIR laser (Laserglow Technologies, Toronto, Ontario, Canada) at 1.5 W/cm^2^ was used for all PTT studies. PBNPs were added to 96-well plates at varied concentrations and irradiated for ten minutes. A thermocouple (Omega Engineering, Stamford, CT) was used to measure the temperature of the wells at one-minute intervals.

### Analysis of PD901 and PBNP-based PTT interaction *in vitro*

The coefficient of drug interaction (CDI) was used to analyze the interaction between PD901 and PBNP-based PTT in treating M2 cells *in vitro*, as previously described^36^. Briefly, M2 cells were subject to varying doses of PD901, PBNP-based PTT, or combination PD901/PTT. The doses of PD901 used were 0.125–100 μM; the doses of PBNP-based PTT were 0.005–0.1 mg/mL irradiated for 10 min with the NIR laser. The doses for the PD901 + PTT combination were the combined doses used for each individual treatment. The viability of the M2 cells at each dose was measured using the ATP-based CellTiter-Glo Luminescent Cell Viability Assay (Promega, Madison, WI). The CDI for a given dose of PD901, PTT, or the PD901/PTT combination was calculated using the formula: CDI = (M2 viability in response to PD901/PTT)/(M2 viability in response to PD901 × M2 viability in response to PTT). A value of CDI < 1 indicated drug synergy, CDI < 0.7 indicated significant drug synergy, CDI = 1 indicated additivity, and CDI > 1 indicated drug antagonism.

### Animals

All animal studies were approved by the Institutional Animal Care and Use Committee (IACUC) of Children’s National Health System, Washington, DC (Protocol #00030571). The studies were conducted in accordance with the approved IACUC guidelines. 4–6 week old female B6129SF1/J mice were purchased from Jackson Laboratory (Bar Harbor, ME). Mice were monitored daily for symptoms of toxicity (e.g. sick mouse posturing, infection, impaired mobility, weight loss, self-mutilation, bleeding). No mice were euthanized due to toxicity symptoms, and no measurable weight loss was recorded. The animals were acclimated for 3–4 days prior to tumor inoculation.

### *In vivo* studies

1 million M2 cells were injected into the backs of B6129SF1/J mice in 100 μL 50% phosphate buffered saline (PBS, Life Technologies, Carlsbad, CA)/50% Matrigel (Corning, Corning, NY). Treatment commenced when tumors reached 10 mm in diameter. Animals were treated in four groups: (1) Untreated (n = 5): animals received no treatment, (2) PTT-treated (n = 5): animals were intratumorally injected with 50 μL of 1 mg/mL PBNPs and irradiated for 10 minutes with 1.5 W/cm^2^ NIR laser, (3) PD901-treated (n = 5): animals were given 5 mg/kg PD901 daily by oral gavage in 0.5% hydroxypropyl methylcellulose and 0.2% Tween80, and (4) PD901 plus PTT combo-treated (n = 5): animals were given both PTT and PD901 treatments as listed above. Animals were euthanized if tumors reached >20 mm in diameter, if their tumors became ulcerated, or if they exhibited signs of distress. Tumors were measured daily by calipers. For histological analysis of the effects of the treatments, tumors were harvested eight hours post treatment (treatments described above; n = 2–3 per group), processed, and stained with hematoxylin and eosin (H&E) for subsequent microscopy.

### Statistical analysis

Statistical analysis was conducted using Prism V5.00 (GraphPad Software, San Diego, CA). Statistical significance between groups in the plots was determined using a two-tailed Student’s *t*-test and values with p < 0.05 qualified as statistically significant and were marked with an asterisk (*) to indicate comparison between two specified groups. ** indicates p-values < 0.01, while *** indicates p-values < 0.001. p-values > 0.05 qualified as not statistically significant. The samples sizes (n) for each group were either ≥3/group or explicitly mentioned for each study. For the animal survival study, a log-rank test was used to determine statistically significant differences in survival between the various groups, (α = 0.05, rejecting the null hypothesis if *x*^2^ > critical value for the test). Survival results were analyzed by generating Kaplan-Meier plots. A p-value < 0.05 was considered statistically significant in this analysis. All relevant p-values are listed in Supplementary Table 1.

## Additional Information

**How to cite this article**: Sweeney, E. E. *et al*. Photothermal therapy improves the efficacy of a MEK inhibitor in neurofibromatosis type 1-associated malignant peripheral nerve sheath tumors. *Sci. Rep.* **6**, 37035; doi: 10.1038/srep37035 (2016).

**Publisher’s note:** Springer Nature remains neutral with regard to jurisdictional claims in published maps and institutional affiliations.

## Supplementary Material

Supplementary Information
