# Supplementary material for: Photothermal therapy improves the efficacy of a MEK inhibitor in neurofibromatosis type 1-associated malignant peripheral nerve sheath tumors
Source: Sci Rep. 2016 Nov 11;6:37035. doi: 10.1038/srep37035 (PMC5105126; doi:10.1038/srep37035)
Supplement: Supplementary Information [file srep37035-s1.pdf]

SUPPLEMENTARY INFORMATION

Photothermal therapy improves the efficacy of a MEK inhibitor in neurofibromatosis type 1-associated malignant peripheral nerve sheath tumors

Elizabeth E. Sweeney[a], Rachel A. Burga[a,c], Chaoyang Li[b], Yuan Zhu[b], Rohan Fernandes*[,a,c,d,e,]

[a] The Sheikh Zayed Institute for Pediatric Surgical Innovation

[b] Center for Cancer and Immunology Research

Children's National Health System, 111 Michigan Ave NW, Washington, DC 20010, USA

[c] Institute for Biomedical Sciences

[d] Department of Radiology

[e] Department of Pediatrics

The George Washington University, 2330 Eye St NW, Washington, DC 20037, USA

Author emails:

Elizabeth E. Sweeney (ESweeney@childrensnational.org); Rachel A. Burga (RBurga@childrensnational.org); Chaoyang Li (CLi@childrensnational.org); Yuan Zhu (YZhu@childrensnational.org)

***Corresponding author**

Rohan Fernandes; Children's National Health System, 111 Michigan Ave NW, Washington, DC 20010, USA; Email: RFernand@childrensnational.org; Phone: (+1) 202-476-5290.

This document contains: 1) Figure S1: H&E staining of the tumors from M2 MPNST-bearing B6129SF1/J mice, **2) Supplementary Table 1:** List of p-values in this study.

SUPPLEMENTARY INFORMATION

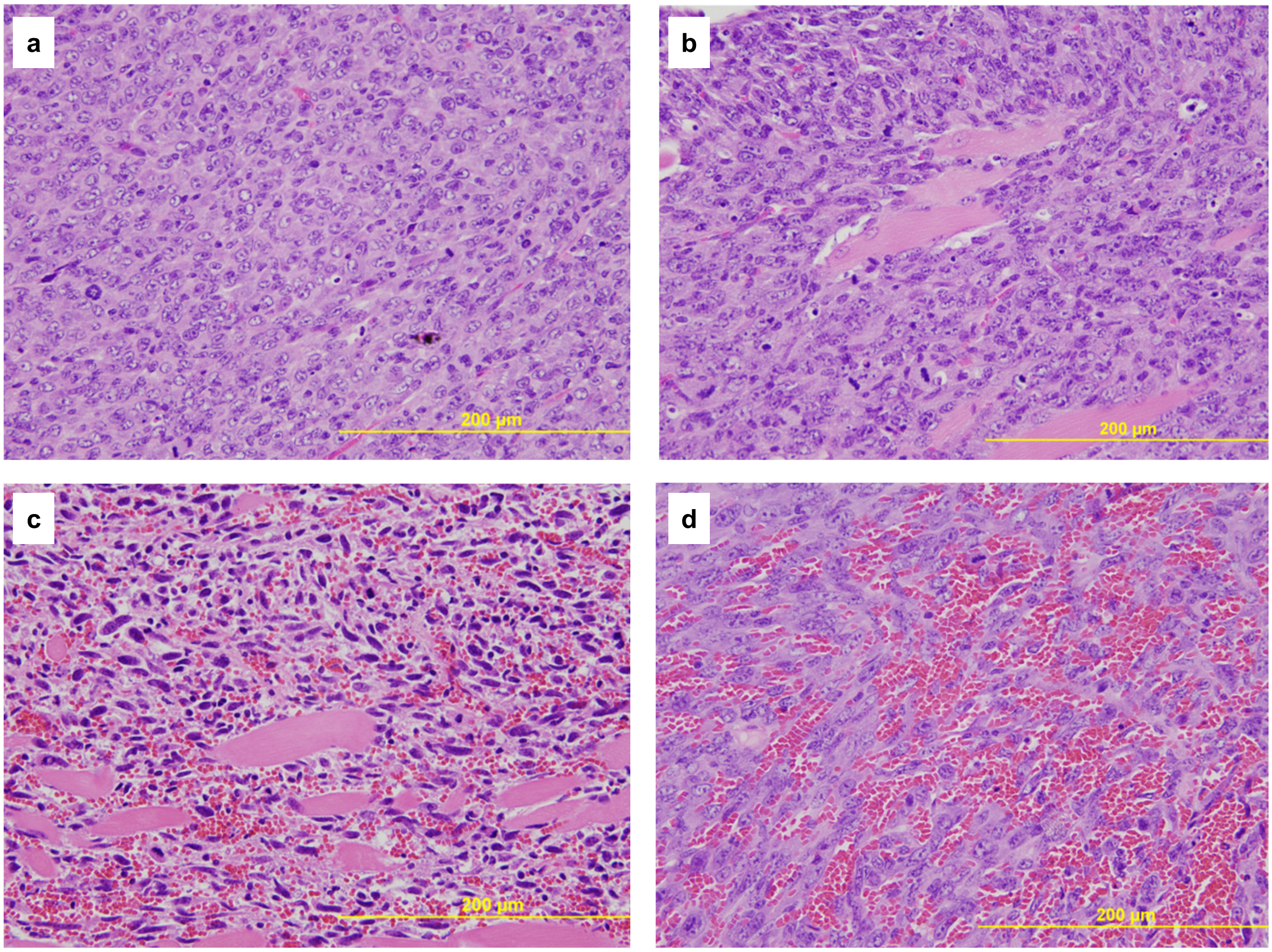

Figure S1. H&E staining of the tumors from M2 MPNST-bearing B6129SF1/J mice that were: a) Untreated, b) PD901-treated: 5 mg/kg PD901, c) PTT-treated: 1.0 mg/mL PBNPs at 1.5 W/cm^2 using an 808 nm NIR laser, or d) PD901 + PTT-treated: both PD901 and PTT treatments using identical treatment conditions for each individual therapy. Tumors were extracted 8 h after treatment.

SUPPLEMENTARY TABLE 1: List of p-values in this study

Figure 1c

Annexin V+ 7AAD-	p-value
Vehicle vs. 0.1 μM PD901	0.004
0.1 μM PD901 vs. 1 μM PD901	0.023
1 μM PD901 vs. 10 μM PD901	0.001
**7AAD+**	**p-value**
Vehicle vs. 0.1 μM PD901	3.11E-04
0.1 μM PD901 vs. 1 μM PD901	0.321
1 μM PD901 vs. 10 μM PD901	0.008

Figure 1d

G0/G1	p-value
Vehicle vs. 0.1 μM PD901	0.018
Vehicle vs. 1 μM PD901	0.001
Vehicle vs. 10 μM PD901	0.001
0.1 μM PD901 vs. 1 μM PD901	0.003
0.1 μM PD901 vs. 10 μM PD901	0.003
1 μM PD901 vs. 10 μM PD901	0.031

Figure 2b

Temperature after 10 minutes	p-value
Vehicle vs. 0.1 mg/mL	0.105
0.01 mg/mL vs. 0.02 mg/mL	0.025
0.02 mg/mL vs. 0.03 mg/mL	0.049
0.03 mg/mL vs. 0.04 mg/mL	0.111
0.04 mg/mL vs. 0.05 mg/mL	0.122
0.05 mg/mL vs. 0.1 mg/mL	1.28E-04
0.1 mg/mL vs. 1.0 mg/mL	1.18E-05

Figure 2c

Viability (+ 1.5 W/cm^2)	p-value
Vehicle vs. 0.01 mg/mL	0.048
Vehicle vs. 0.02 mg/mL	3.55E-05
Vehicle vs. 0.03 mg/mL	3.54E-06
Vehicle vs. 0.04 mg/mL	3.01E-04
Vehicle vs. 0.05 mg/mL	4.80E-05

Figure 2d

Annexin V+ 7AAD-	p-value
0.03 mg/mL vs. Vehicle	0.002
0.03 mg/mL vs. 1.5 W/cm^2	0.002
0.03 mg/mL vs. 0.01 mg/mL	0.007
0.03 mg/mL vs. 0.05 mg/mL	0.001

7AAD+	p-value
0.05 mg/mL vs. Vehicle	9.28E-05
0.05 mg/mL vs. 1.5 W/cm^2	9.35E-05
0.05 mg/mL vs. 0.01 mg/mL	0.039
0.05 mg/mL vs. 0.03 mg/mL	0.001

Figure 3a

Viability	p-value
LOW Combo vs. LOW PD901	0.029
LOW Combo vs. LOW PTT	0.002
HIGH Combo vs. HIGH PD901	0.009
HIGH Combo vs. HIGH PTT	0.024

Figure 4b

Survival	p-value
Untreated vs. PTT	0.220
Untreated vs. PD901	0.003
Untreated vs. Combo	0.003
PTT vs. PD901	0.400
PTT vs. Combo	0.006
PD901 vs. Combo	0.003
